# Sustained Benefits of a Multidomain Lifestyle Intervention in Multiple Sclerosis in the LIMS Study: A 24-Month Follow-Up

**DOI:** 10.1212/WNL.0000000000218542

**Published:** 2026-09-24

**Authors:** Arianne S. Gravesteijn, Ilse M. Nauta, Meike Stob, Nathalie Wilmsen, Emma Coles, Vincent de Groot, Zoe L. E. van Kempen, Joep Killestein, Bob W. Van Oosten, Eva M. Strijbis, Bernard M.J. Uitdehaag, Brigit A. de Jong

**Affiliations:** 1 Amsterdam UMC Location Vrije Universiteit Amsterdam, Rehabilitation Medicine, the Netherlands;; 2 Amsterdam Neuroscience, Neuroinfection and Neuroinflammation, the Netherlands;; 3 Amsterdam Movement Sciences, Rehabilitation and Development, the Netherlands;; 4 MS Center Amsterdam, the Netherlands;; 5 Amsterdam UMC Location Vrije Universiteit Amsterdam, Neurology, the Netherlands;; 6 Voeding Leeft, Amsterdam, the Netherlands; and; 7 Amsterdam Public Health, Quality of Care, the Netherlands.

## Abstract

**Background and Objectives:**

Multiple sclerosis (MS) often leads to physical, cognitive, and psychological impairments substantially affecting daily functioning and quality of life. Because current disease-modifying therapies do not fully prevent disease progression, interest has grown in the potential role of modifiable lifestyle factors. We evaluated (1) whether effects on patient-reported outcomes were sustained up to 24 months and (2) long-term changes in lifestyle behaviors after completion of the lifestyle in MS program.

**Methods:**

People with MS participated in a 3-month multimodal lifestyle program (i.e., Mediterranean-style diet, physical activity, and sleep and stress), followed by a 21-month maintenance phase. Patient-reported outcomes were assessed 3 months before the intervention, at baseline, postintervention, and after 6, 12, 18, and 24 months. The primary outcome was MS-related impact on daily functioning (MS Impact Scale–29 [MSIS-29]). Secondary outcomes included quality of life, general health, and MS-related symptoms. Longitudinal changes were analyzed using piecewise mixed-effects models, and individual-level change was evaluated using proportions exceeding the smallest detectable change (SDC).

**Results:**

In total, 659 participants were included (mean age 46 years, SD 11; 84% female). During the intervention phase, MSIS-29 physical and psychological scores significantly decreased by approximately 0.8 and 1.1 points per month, respectively, followed by attenuation during long-term follow-up. This was reflected by within-group changes of −2.7 (−3.7; –1.8) and −3.7 (−4.8; –1.6) points after the intervention and −0.8 (−1.7; 0.3) and −3.4 (−4.6; –2.3) point at month 24, remaining significantly improved or stable relative to baseline. Only a small proportion of participants exceeded the SDC for MSIS-29 (approximately 4%–7%). Trajectories were broadly similar across other patient-reported outcomes, with modest short-term improvements and subsequent stabilization. Most pronounced effects were found for dietary adherence and body mass index, which improved substantially during the intervention and remained partially sustained during follow-up. No significant changes were observed in physical activity adherence.

**Discussion:**

In this large, real-world study, a digital multimodal lifestyle intervention was associated with modest, mainly short-term improvements in patient-reported outcomes and more pronounced individual-level changes in dietary behavior and body composition. These findings support a complementary role for lifestyle interventions in long-term MS management.

**Trial Registration Information:**

The study was registered at ClinicalTrials.gov (Identifier: NCT05402501; classic.clinicaltrials.gov/ct2/show/NCT05402501).

## Introduction

Despite decades of substantial therapeutic advancement, many people with multiple sclerosis (MS) continue to face persistent symptoms, accumulating disability, and reduced quality of life.^1^ Although disease-modifying therapies (DMTs) have transformed the management of MS by targeting focal inflammatory activity, their capacity to fully arrest neurodegeneration remains limited.^1^ This therapeutic gap has prompted growing scientific interest in the role of modifiable lifestyle factors as potential adjuncts to pharmacologic treatment.^2^

A healthy lifestyle is not defined by a single habit but rather by the interaction of multiple domains, including diet, physical activity, sleep, and stress regulation, all of which may influence MS.^3^ Emerging evidence suggests that behaviors, such as regular physical activity, balanced nutrition, restorative sleep, and psychological well-being, may affect not only symptom burden but also biological processes relevant to disease progression.^2^

The Mediterranean diet, characterized by high intake of fruits, vegetables, olive oil, fish, and nuts, has been associated with reduced disease severity and may exert anti-inflammatory effects.^4-6^ Regular physical activity is associated with improved symptoms and functional capacity and may ameliorate pathophysiologic hallmarks of MS.^7,8^ Sleep problems and stress have been less extensively studied but do correlate with quality of life, fatigue, and may negatively affect disease burden.^9,10^ Multimodal interventions that target several of these factors simultaneously are being explored for their synergistic effects on both symptoms and underlying pathophysiology and may offer greater benefits than targeting each domain in isolation.^11,12^

A critical consideration in the implementation of lifestyle interventions is accessibility. For people with MS, mobility impairments, travel distance, and logistical challenges often pose significant barriers.^13^ Advances in digital health offer promising solutions for delivering personalized interventions directly within the home environment.^14^ In addition, remote formats may also support long-term engagement and adherence, fostering sustainable lifestyle changes essential in chronic disease management. Nevertheless, both long-term lifestyle changes and the durability of clinical effects of digital lifestyle interventions remain underexplored.^12^

An MS-specific, digital multidomain lifestyle intervention (LIMS) was developed, targeting diet, physical activity, sleep, and stress management.^15^ The LIMS program demonstrated short-term benefits on the impact of MS, daily functioning, quality of life, and general health determinants.^15^ Here, we aim to evaluate: (1) whether the effects on patient-reported outcome measures (PROMs) and self-reported anthropometric measures, including the impact of MS, quality of life, general health, and MS-related symptoms are sustained up until 24 months, and (2) long-term changes in behavioral factors after completing the initial LIMS program.

## Methods

### Study Design

The LIMS study is a longitudinal prospective study designed to assess the effects of a, multidomain lifestyle intervention in people with MS.^15^ The study included 3 distinct phases: a 3-month run-in period (timepoint (T)-3 to T0), a 3-month intensive intervention phase (T0 to T3), a 21-month maintenance phase with assessments at T3, T6, T12, T18, and T24. During the run-in phase, participants continued their usual daily routines and did not receive any lifestyle guidance or monitoring. This period served as a control phase, enabling within-subject comparisons across all study phases.

Ethical approval was waived by the Medical Ethics Review Committee of Amsterdam University Medical Center, location VUmc (reference: 2021.0089). The LIMS study was prospectively registered at ClinicalTrials.gov (identifier: NCT05402501).

### Participants

People with MS were recruited between 2021 and 2022 through the Dutch National MS Foundation, MS Centrum Amsterdam, and “Nutrition Lives.” Interested individuals could apply through an online registration form.^15^

To be eligible, participants had to meet the following criteria: confirmed MS diagnosis according to the McDonald 2017 criteria,^16^ age 18–75 years, and the ability to complete online questionnaires and independently (or with some assistance) shop for groceries and prepare program meals. Exclusion criteria included inability to speak or read Dutch, adherence to a vegan diet, unwillingness to consume fish, body mass index (BMI) <18.5 or >35 kg/m^2^, pregnancy or lactation, diagnosis of an eating disorder, a diagnosed psychiatric illness (according to DSM-V), inflammatory bowel disease (e.g., Crohn disease or ulcerative colitis), type 1 or type 2 diabetes, malignancy, moderate-to-severe cardiovascular disease, history of bariatric surgery, participation in a lifestyle program, or treatment by a dietitian or lifestyle coach.

All participants provided informed consent before enrollment. The study was conducted in accordance with the Declaration of Helsinki.

### Outcome Measures

At the run-in assessment (T-3), we collected demographic characteristics, clinical information (MS diagnosis obtained from medical records), and self-reported health indicators (height, smoking status, alcohol consumption, vegetarian status, supplement use, and comorbidities).

MS impact, lifestyle factors, general health, and MS-related symptoms were evaluated at all study time points (T-3, T0, T3, T6, T12, T18, and T24).

#### MS Impact

The primary outcome was change in the impact of MS on daily functioning, divided into a physical and a psychological domain (i.e., MS Impact Scale [MSIS-29]).^17^ A 29-item PROM comprising 20 physical and 9 psychological items. Scores of both subscales were converted to a 0–100 scale, with lower scores indicating lower impact. To contextualize the clinical relevance of observed changes in the primary outcome, published minimal important change (MIC) thresholds were used (physical subscale, MIC thresholds of 8.0^18^ and 9.1 points^19^; psychological subscale 5.5 points^19^).

#### Quality of Life and General Health

General health was assessed using the 12-item Short Form Health Survey (SF-12), evaluating physical and mental health–related quality of life.^20^ Participants self-reported anthropometrics (weight, height, and waist circumference) using personal scales and measuring tapes; BMI was subsequently calculated (kg/m^2^). Stool quality was assessed using the Bristol Stool Chart.^21^

#### MS-Related Symptoms

Anxiety, depression, fatigue, and cognitive complaints were assessed with the validated PROMs Hospital Anxiety and Depression Scale,^22^ the Checklist Individual Strength–20-R (CIS-20r) fatigue subscale,^23^ and the MS Neuropsychological Screening Questionnaire—Patient version,^24^ respectively.

#### Long-Term Lifestyle Changes and Adherence

Lifestyle changes were evaluated across the 4 domains: diet, physical activity, stress, and sleep. Adherence to the Mediterranean diet was measured using a modified 17-item Mediterranean Diet Score.^25^ Modifications include splitting the single meat item into processed meat, unprocessed red meat, and poultry; removing the sofrito item due to limited cultural relevance in the Netherlands; and adding 3 items reflecting Dutch dietary patterns: number of eating moments per day, dairy fat content preference, and wholegrain vs refined grain consumption. Physical activity was assessed using the questionnaire from the National Institute for Public Health and the Environment (RIVM).^26,27^ Perceived levels of stress were quantified using the 14-item Perceived Stress Scale,^28^ while sleep quality was assessed using the Medical Outcomes Study Sleep Scale, sleep index II.^29^

Long-term adherence and lifestyle changes were monitored throughout the 21-month maintenance phase (T3–T24). We assessed the number of sessions attended. Sustained physical activity was defined as adherence to Dutch physical activity guidelines (≥150 minutes of moderate activity; ≥2 times resistance exercises).^26^ For diet (total diet score), a change exceeding the smallest detectable change (SDC) was considered indicative of long-term adherence or improvement.^30^ For sleep and stress, no direct measures for adherence were available.

### Lifestyle Intervention

The LIMS program is a comprehensive 2-year fully digital intervention targeting 4 key lifestyle pillars: a Mediterranean-style diet, supported by recipes and 28-day meal plans; physical activity aimed to increase activity levels through individualized activity planning; stress management incorporating breathing techniques, mindfulness, gratitude exercises, and positive psychology strategies; and psychoeducation on sleep hygiene and practical strategies were provided to improve sleep quality, grounded in the I-Change model theory with behavior change techniques applied across all lifestyle pillars.^31^ The program consisted of an initial 3-month intensive intervention phase followed by a 21-month maintenance phase. Throughout the entire program, participants had continuous access to an interactive online platform. For a detailed description of the program (eAppendix 1) TIDieR checklist.

The intensive 3-month intervention phase consisted of main sessions (∼100 participants) and themed sessions (∼25 participants), both online. The 3 main sessions of 5 hours focused sequentially on nutrition, physical activity, relaxation, and sleep, complemented by 5 1-hour themed sessions in smaller groups addressing nutrition, personal development, reflection on progress, physical activity, relaxation, and personal growth. Sessions were facilitated by a moderator and delivered by lifestyle coaches, dietitians, and domain-specific experts in physical activity, relaxation, and sleep.

After the intensive phase, participants entered a 21-month maintenance phase. Frequency of meetings decreased to 4 webinars about nutrition, physical activity, relaxation, and sleep and 5 1-hour theme coaching sessions to help participants embed lifestyle changes into their daily lives, maintain engagement, and refresh knowledge.

### Statistical Analyses

All statistical analyses were performed using RStudio version 2.4.1.

To examine the long-term effects of the LIMS study, we applied piecewise linear mixed-effect models.^32^ To characterize differential trajectories across study phases, we fitted a three-piece linear spline with knots at 0 and 3 months, representing the run-in period (−3 to 0 months), intensive intervention phase (0–3 months), and maintenance phase (3–24 months). The model included fixed effects for time, hinge terms at 0 and 3 months, allowing slopes to differ between phases. Random intercepts were included for participants to account for within-subject correlation. Phase-specific slopes and contrasts between slopes were derived using linear combinations of fixed effects, with 95% CIs calculated through the delta method. The within-person analytic approach inherently controlled for all time-invariant confounders by treating each participant as their own control.^32^

Complementary linear mixed-effects models with time treated as a categorical factor were fitted to estimate model-based mean outcomes at each measurement occasion and to identify at which follow-up timepoints outcomes differed from baseline (0 months).

The SDC at an individual level was calculated using the run-in period to estimate the proportion of participants exhibiting a detectable change at individual level at each timepoint (eMethods: calculations).

This three-step analytical approach, comprising piecewise mixed-effects models, linear mixed-effects models, and proportion analyses, was also applied to the lifestyle factors.

### Data Availability

The data of this research can be requested from the corresponding author, immediately following publication and with no end date, by any qualified researchers who engage in rigorous independent scientific research.

## Results

### Participants

A total of 659 people with MS were included. Participant attrition increased over time (Figure 1). The most commonly reported reasons for dropout were personal reasons, unmet expectations, program intensity, and health-related issues; however, reasons were frequently unreported. Baseline characteristics are summarized in Table 1. Participants had a mean age of 46.2 years (SD 10.7), 84% were female, and 71% had relapsing-remitting MS.

**Figure 1:**
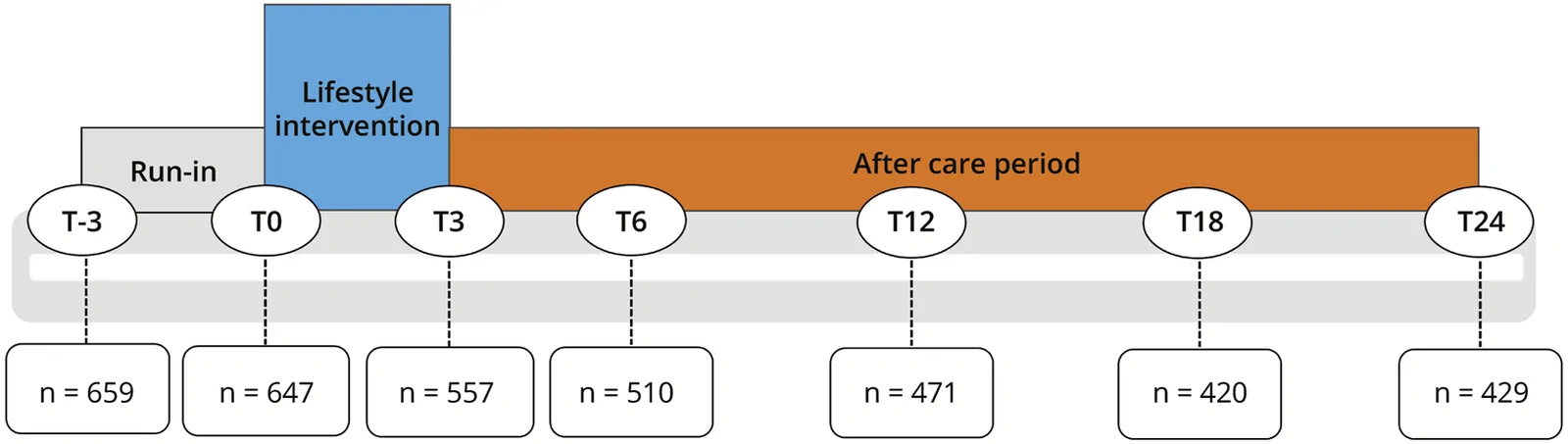
Flowchart LIMS Project Run-In Phase (T-3 and T0), Short-Term Follow-Up (T3 and T6), and Long-Term Follow-Up (T12, T18, and T24) LIMS = Lifestyle in Multiple Sclerosis.

**Table 1:** Baseline Characteristics of the Study Population

Baseline characteristics	Complete sample (n = 659)	Sufficient follow-up (n = 500)	Insufficient follow-up (n = 159)
Demographics			
Age (y), mean (SD)	46.2 (10.7)	**46.7 (10.4)**	**44.6 (11.7)**
Sex (women), n (%)	559 (84)	425 (85)	134 (84)
Education, n (%)			
Low	23 (3)	15 (3)	8 (5)
Middle	490 (74)	369 (74)	121 (76)
High	146 (22)	116 (23)	30 (19)
Work, n (%)			
Paid work	282 (43)	211 (42)	71 (45)
Unpaid work	14 (2)	14 (3)	0 (0)
Paid work, partially on sick leave	67 (10)	52 (1)	15 (9)
Paid work, fully on sick leave	35 (5)	28 (6)	7 (4)
Permanently disabled for work	188 (29)	144 (29)	44 (27)
Unemployed/job seeking	11 (2)	8 (2)	3 (2)
Student	11 (2)	6 (1)	5 (3)
Homemaker	33 (5)	22 (4)	11 (7)
Retired	18 (3)	15 (3)	3 (2)
Family situation, n (%)			
Living alone	91 (14)	68 (14)	23 (14)
Cohabiting without children	167 (25)	134 (27)	33 (33)
Cohabiting with children	339 (51)	259 (52)	80 (50)
Other	62 (9)	39 (8)	23 (14)
Disease-related characteristics			
MS subtype (RRMS/SPMS/PPMS/not defined), n (%)	466(71)/62(9)/79(12)/40(6)	355(71)/42(8)/64 (13)/39(8)	111(70)/20(13)/15(9)/1 (8)
Symptom duration (y), median (IQR)	10.9 (5.0; 19.4)	10.9 (5.4; 19.3)	11.3 (4.3; 20.0)
Diagnosis duration (y), median (IQR)	7.2 (2.5; 13.8)	7.2 (2.5; 13.8)	7.2 (2.5; 13.6)
DMT (yes), n (%)	422 (64)	324 (65)	98 (62)
Self-reported general health			
BMI (kg/m^2^), mean (SD)^a^	25.5 (4.5)	25.6 (4.5)	25.5 (4.3)
Underweight, n (%)	6 (1)	5 (1)	1 (1)
Normal, n (%)	333 (51)	249 (50)	84 (53)
Overweight, n (%)	204 (31)	156 (31)	48 (30)
Obese, n (%)	115 (17)	89 (18)	26 (16)
Waist (cm), mean (SD)^b^	91.7 (13.2)	91.9 (13.5)	90.8 (12.5)
Low risk, n (%)	183 (28)	142 (29)	41 (26)
High risk, n (%)	150 (23)	**102 (20)**	**48 (31)**
Very high risk, n (%)	321 (49)	254 (51)	67 (43)
Smoking (yes, quit, never), %	25(3)/276(42)/358(54)	**15(3)/**202(40)/283(57)	**10 (6)**/74(47)/75(47)
Alcohol (never, ≤2 d/wk, ≥3 d/wk), n (%)	235(36)/346(53)/68(10)	178(36)/257(51)/65(13)	57(36)/89(56)/13(8)
Vegetarian (yes), n (%)	59 (9)	44 (9)	15 (9)
Self-reported comorbidities (yes), n (%)^c^	202 (31)	149 (30)	53 (33)

Abbreviations: BMI = Body mass index; DMT = Disease-modifying therapy; IQR = Interquartile range; MS = multiple sclerosis; PPMS = Primary progressive multiple sclerosis; RRMS = Relapsing-remitting multiple sclerosis; SPMS = Secondary progressive multiple sclerosis.

a BMI: underweight <18.5, normal 18.5–24.9, overweight 25.0–29.9, and obese >30.

b Women: low risk <80 cm, high risk 80–88 cm, and very high risk >88 cm; Men: low risk <94 cm, high risk 94–102 cm, and very high risk >102 cm.

c The individual supplement types and comorbidities do not add up to 100% because participants could report multiple supplements or multiple comorbidities simultaneously, or none at all, a detailed list of comorbidities is provided elsewhere.^15^ Bold-italic represents significant group difference (*p* < 0.05).

Selective attrition analyses showed that participants with sufficient follow-up were slightly older at baseline (46.7 ± 10.4 vs 44.6 ± 11.7 years, *p* = 0.03), had a lower prevalence of high-risk waist circumference (20% vs 31%), and were less frequently smokers (3% vs 6%).

Attendance across intensive phase and maintenance phase sessions, excluding dropouts at each time point, is summarized in Table 2. Attendance was high during the intensive intervention phase (95% introductory session; 93% ≥ 2 main meetings; 68% ≥ 4 thematic sessions) and declined during the maintenance phase, from approximately 50%–56% at the first coaching and webinar sessions to around 20% at the final sessions.

**Table 2:** Attendance Rates for LIMS Sessions for Participants Who Provided Information at Each Timepoint

LIMS intervention phase (T0-T3)			
Components T3	Sessions attended	n = 557	%
Introduction	1 session	529	95.0
	0 sessions	27	4.3
	Missing	1	0.2
Main meetings	3 sessions	384	68.9
	2 sessions	135	24.2
	1 session	33	5.9
	0 sessions	3	0.5
	Missing	2	0.4
Theme sessions	5 sessions	194	34.8
	4 sessions	187	33.6
	3 sessions	38	6.8
	2 sessions	98	17.6
	1 session	22	3.9
	0 sessions	16	2.9
	Missing	2	0.4

21-mo follow-up maintenance program (T3-T24)			
Components T6	Sessions attended	n = 510	%
Coaching session 1	1 session	256	50.2
	0 sessions	245	48.0
	Missing	9	1.8
Booster webinar diet	1 session	285	55.9
	0 sessions	216	42.4
	Missing	9	1.8

Components T12	Sessions attended	n = 471	%
Coaching session 2	1 session	210	44.6
	0 sessions	256	54.4
	Missing	5	1.1
Booster webinar physical activity	1 session	125	26.5
	0 sessions	341	72.4
	Missing	5	1.1

Components T18	Sessions attended	n = 420	%
Coaching session 3	1 session	123	29.3
	0 sessions	291	69.3
	Missing	6	1.4
Booster webinar stress	1 session	99	23.6
	0 sessions	315	75.0
	Missing	6	1.4

Components T24	Sessions attended	n = 429	%
Coaching session 4	1 session	111	25.9
	0 sessions	309	72.0
	Missing	9	2.1
Booster webinar sleep	1 session	96	22.4
	0 sessions	323	75.3
	Missing	10	2.3
Coaching session 5	1 session	87	20.3
	0 sessions	332	77.4
	Missing	10	2.3

Abbreviation: LIMS = Lifestyle in Multiple Sclerosis.

### Long-Term Intervention Effects

The results from the piecewise mixed-effects models are presented in Table 3, with complementary linear mixed-effects models (time as categorical) in eTable 1 and proportions exceeding the SDC in eTable 2. Sensitivity analyses restricted to participants with sufficient follow-up are presented in eAppendix 2 and eTables 3 and 4 and SDC details in eTable 5. Model-based trajectories are presented in Figure 2 (primary outcome) and eFigures 1–11 (secondary outcomes).

**Table 3:** Piecewise Mixed Model

Phase	Slope (point/month)	95% CI		*p* Value	Interpretation	Contrast	Δ slope (points/month)	95% CI		*p* Value
		Lower	Upper					Lower	Upper	
Multiple sclerosis impact scale–physical										
Run-in	−0.24	−0.54	0.05	0.105	Stable	Run-in vs intervention	−0.60	−1.09	−0.10	0.018
Intervention	−0.84	−1.11	−0.57	<0.001	Improvement	Intervention vs follow-up	0.92	0.63	1.21	<0.001
Follow-up	0.08	0.04	0.13	<0.001	Deterioration					
Multiple sclerosis impact scale—psychological										
Run-in	−0.60	−0.95	−0.26	0.001	Improvement	Run-in vs intervention	−0.48	−1.06	0.10	0.104
Intervention	−1.09	−1.40	−0.77	<0.001	Improvement	Intervention vs follow-up	+1.08	0.74	1.43	<0.001
Follow-up	−0.00	−0.05	0.05	0.941	Stable					
SF12 physical										
Run-in	0.27	0.10	0.44	0.002	Improvement	Run-in vs intervention	−0.12	−040	0.17	0.433
Intervention	0.15	−0.00	0.31	0.054	Stable	Intervention vs follow-up	−0.17	−0.34	0.00	0.052
Follow-up	−0.01	−0.04	0.01	0.281	Stable					
SF12 mental										
Run-in	−0.10	−0.28	0.08	0.262	Stable	Run-in vs intervention	0.37	0.07	0.67	0.016
Intervention	0.27	0.11	0.43	0.001	Improvement	Intervention vs follow-up	−0.28	−0.46	−0.11	0.002
Follow-up	−0.01	−0.04	0.01	0.299	Stable					
Body mass index (kg/m^2^)										
Run-in	0.03	−0.01	0.07	0.119	Stable	Run-in vs intervention	−0.28	−0.35	−0.21	<0.001
Intervention	−0.25	−0.28	−0.21	<0.001	Improvement	Intervention vs follow-up	+0.28	0.23	0.31	<0.001
Follow-up	0.02	0.02	0.03	<0.001	Deterioration					
Waist circumference (cm)										
Run-in	0.06	−0.12	0.24	0.515	Stable	Run-in vs intervention	−0.95	−1.26	−0.64	<0.001
Intervention	−0.89	−1.06	−0.72	<0.001	Improvement	Intervention vs follow-up	0.95	0.77	1.13	<0.001
Follow-up	0.06	0.03	0.09	<0.001	Deterioration					

Phase	Slope (point/month)	95% CI		*p* Value	Interpretation	Contrast	Δ slope (points/month)	95% CI		*p* Value
		Lower	Upper					Lower	Upper	
Bristol stool chart^a^										
Run-in	1.02	0.95	1.09	0.595	Stable	Run-in vs intervention	1.11	0.98	1.24	0.102
Intervention	1.12	1.06	1.20	0.003	Increased odds of higher stool category	Intervention vs follow-up	0.89	0.82	0.94	<0.001
Follow-up	0.99	0.98	1.00	0.060	Stable					
Hospital anxiety and depression scale—anxiety										
Run-in	−0.06	−0.13	0.01	0.070	Stable	Run-in vs intervention	−0.04	−0.15	0.08	0.526
Intervention	−0.10	−0.16	−0.04	0.002	Improvement	Intervention vs follow-up	0.09	0.03	0.16	0.007
Follow-up	−0.01	−0.02	0.00	0.254	Stable					
Hospital anxiety and depression scale—depression										
Run-in	0.01	−0.06	0.08	0.790	Stable	Run-in vs intervention	−0.16	−0.28	−0.04	0.008
Intervention	−0.15	−0.22	−0.09	<0.001	Improvement	Intervention vs follow-up	0.16	0.09	0.23	<0.001
Follow-up	0.01	0.00	0.02	0.010	Deterioration					
CIS20r fatigue subscale										
Run-in	−0.28	−0.52	−0.05	0.017	Improvement	Run-in vs intervention	−0.49	−0.88	−0.09	0.015
Intervention	−0.77	−0.98	−0.56	<0.001	Improvement	Intervention vs follow-up	0.84	0.61	1.07	<0.001
Follow-up	0.07	0.03	0.10	<0.001	Deterioration					
Multiple Sclerosis Neuropsychological Screening Questionnaire—patient version										
Run-in	−0.04	−0.19	0.11	0.590	Stable	Run-in vs intervention	−0.05	−0.31	0.20	0.690
Intervention	−0.09	−0.23	0.05	0.189	Stable	Intervention vs follow-up	0.07	−0.08	0.22	0.355
Follow-up	−0.02	−0.04	0.00	0.051	Stable					

Abbreviations: CIS-20r = Checklist Individual Strength-20-r; SF-12 = Short Form Health Survey—12-items.

a The Bristol Stool Chart is a categorical variable; therefore, ordinal mixed models were used and outcomes are presented in odds ratios.

**Figure 2:**
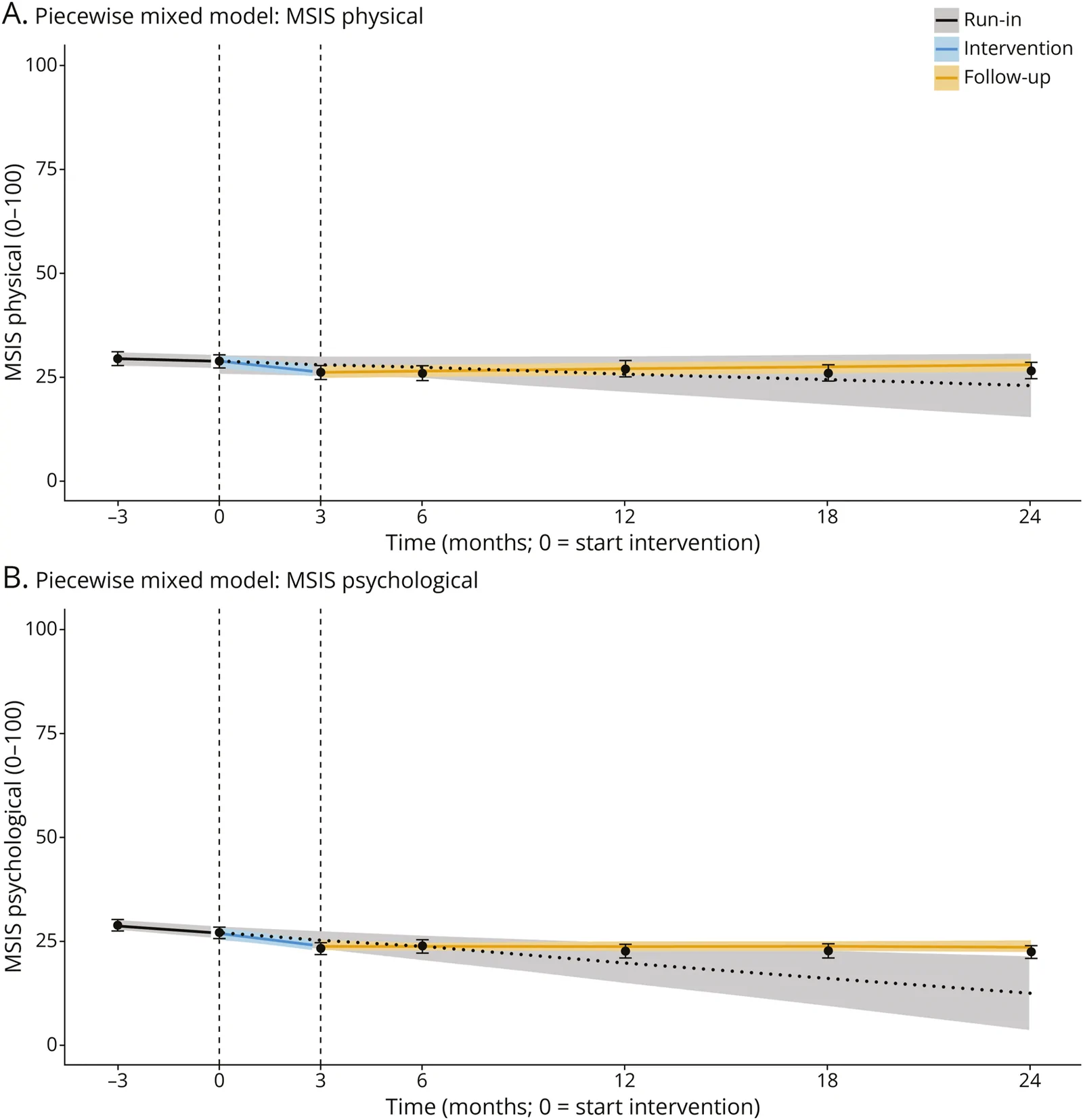
Piecewise Mixed Model MSIS From −3 to 0 in black the run-in phase, from 0 to 3in blue the intervention phase, and from 3 to 24 the follow-up period in orange. The dotted line with gray error bands the counterfactual projection. MSIS = Multiple Sclerosis Impact Scale.

#### MS Impact

During the run-in period (−3 to 0 months), MSIS physical scores remained stable, with a nonsignificant decline of 0.24 points per month (95% CI –0.54 to 0.05; *p* = 0.105), whereas MSIS psychological scores showed a modest but significant improvement (−0.60 points per month, 95% CI –0.95 to −0.26; *p* = 0.001).

During the intensive intervention phase (0–3 months), both subscales demonstrated a steeper improvement. MSIS physical scores declined by 0.84 points per month (95% CI –1.11 to −0.57; *p* < 0.001), and MSIS psychological scores declined by 1.09 points per month (95% CI –1.40 to −0.77; *p* < 0.001). The change in slope from run-in to intensive intervention phase was statistically significant for the physical subscale (Δ −0.60 points/month, 95% CI –1.09 to −0.10; *p* = 0.018), but not for the psychological subscale (Δ −0.48 points/month, 95% CI -1.06 to 0.10; *p* = 0.104).

During the maintenance phase (3–24 months), trajectories diverged between subscales. For MSIS physical, the slope changed relative to the intensive intervention phase, showing a small but significant increase of 0.08 points per month (95% CI 0.04–0.13; *p* < 0.001), indicating attenuation of the initial improvement. For MSIS psychological, the maintenance phase slope flattened and did not differ significantly from zero (−0.00 points per month, 95% CI –0.05 to 0.05; *p* = 0.941), reflecting stabilization. Phase-specific slopes and slope contrasts are presented in Table 3.

Despite attenuation of slopes during the maintenance phase, MSIS physical scores remained significantly below baseline at 6, 12, and 18 months after the intensive intervention, whereas MSIS psychological scores remained significantly below baseline at all maintenance phase timepoints (6, 12, 18, and 24 months; Table 3).

A decrease exceeding the SDC was considered indicative of a real, detectable change (MSIS-physical: 19.20 points; MSIS-psychological: 24.43 points). Table 4 presents the proportion of participants demonstrating such a change relative to the start of intervention at 3, 6, 12, 18, and 24 months. Across follow-up, between 3.8% and 6.4% of participants exceeded the SDC for MSIS physical, and between 4.9% and 7.5% exceeded the SDC for MSIS psychological.

**Table 4:** Piecewise Mixed Models Lifestyle Factors

Phase	Slope (point/month)	95% CI		*p* Value	Interpretation	Contrast	Δ slope (points/month)	95% CI		*p* Value
		Lower	Upper					Lower	Upper	
Diet adherence										
Run-in	0.01	−0.05	0.07	0.687	Stable	Run-in vs intervention	1.07	0.97	1.17	<0.001
Intervention	1.08	1.03	1.13	<0.001	Improvement	Intervention vs follow-up	−1.15	−1.21	−1.09	<0.001
Follow-up	−0.06	−0.08	−0.06	<0.001	Deterioration					
Adherence Dutch physical activity guidelines^a^										
Run-in	0.95	0.86	1.05	0.312	Stable	Run-in vs intervention	1.03	088	1.21	0.688
Intervention	0.98	0.90	1.07	0.719	Stable	Intervention vs follow-up	1.02	0.93	1.12	0.676
Follow-up	1.00	0.99	1.02	0.561	Stable					
Perceived Stress Scale										
Run-in	−0.22	−0.39	−0.05	0.011	Improvement	Run-in vs intervention	−0.12	−0.41	0.16	0.403
Intervention	−0.34	−0.49	−0.18	<0.001	Improvement	Intervention vs follow-up	0.34	0.17	0.51	<0.001
Follow-up	0.00	−0.02	0.03	0.783	Stable					
MOS-SS Sleep Index II										
Run-in	0.07	−0.24	0.39	0.643	Stable	Run-in vs intervention	−1.56	−2.10	−1.03	<0.001
Intervention	−1.49	−1.78	−1.20	<0.001	Improvement	Intervention vs follow-up	1.58	1.27	1.89	<0.001
Follow-up	0.09	0.05	0.14	<0.001	Stable					

Abbreviation: MOS-SS = Medical Outcomes Study Sleep Scale.

a Adherence to Dutch physical activity guidelines is a binominal outcome; therefore, logistic mixed models were used, and outcomes are presented in odds ratios.

#### Quality of Life

The SF-12 physical subscale showed a modest improvement during the run-in period but no significant changes during intensive intervention or maintenance phase. By contrast, the mental subscale improved significantly during the intensive intervention phase and subsequently stabilized, with a significant attenuation of slope during the maintenance phase. Relative to baseline, improvements were confined to the intervention period for the mental subscale. Only a small proportion of participants exceeded the SDC (physical: 3.6%–5.3%; mental: 0.4%–1.4%).

#### General Health

BMI and waist circumference remained stable during run-in, improved during the intensive intervention phase, and partially deteriorated during the maintenance phase. Nonetheless, both measures remained significantly below baseline at all follow-up time points. A detectable improvement in BMI was observed in 20% of participants postintervention and 15% at 24 months, whereas detectable changes in waist circumference were less frequent (6.9%–9.5%). Stool consistency showed increased odds of looser stools during the intensive intervention, with effects attenuating during the maintenance phase.

#### MS-Related Symptoms

Anxiety and depression scores improved modestly during the intensive intervention phase, with a significant slope change for depression only. During the maintenance phase, anxiety stabilized while depression showed slight deterioration, indicating partial loss of intervention effects. Fatigue improved during both the run-in and intensive intervention phases, with the largest improvement during the intensive intervention phase, followed by gradual attenuation during the maintenance phase; however, scores remained below baseline at most follow-up assessments. Cognitive complaints remained largely stable across all phases, with only small, transient improvements. Across MS-related symptoms, the proportion of participants exceeding the SDC was low (generally <10%).

### Long-Term Lifestyle Changes

Changes in lifestyle factors were evaluated using piecewise mixed-effects models (Table 4), complementary linear mixed-effects models with time as a categorical factor (eTable 6), and proportions exceeding the (SDC; eTable 7). Trajectories of continuous outcomes are shown in eFigures 1–11.

Mediterranean diet adherence was stable during the run-in period, improved substantially during the intensive intervention phase, and partially declined during the maintenance phase, although scores remained significantly improved relative to baseline at all follow-up assessments. The proportion of participants exceeding the SDC decreased from 53% postintervention to 27% at 24 months, indicating attenuation over time.

Physical activity adherence remained unchanged throughout the study, with approximately 30% of participants meeting Dutch physical activity guidelines at all time points.

Perceived stress improved during the run-in and intensive intervention phases, followed by stabilization and partial deterioration during the maintenance phase. Despite this attenuation, stress levels remained significantly lower than baseline across all assessments. Only a small proportion of participants exceeded the SDC (4.6%–7.6%).

Sleep quality remained stable during the run-in period, improved during the intensive intervention phase, and stabilized during the maintenance phase. Sleep scores remained significantly improved relative to baseline at all follow-up time points, with 10% of participants exceeding the SDC postintervention and 6% at 18 months.

## Discussion

This long-term follow-up study evaluated the effects of the LIMS program, a comprehensive lifestyle intervention targeting diet, physical activity, stress, and sleep in people with MS. First, we assessed sustained effects on PROMs, including MS impact on daily functioning, quality of life, general health, and MS-related symptoms. Second, we examined long-term changes in lifestyle behaviors. Together, the findings highlight both the potential and the limitations of lifestyle interventions in MS.

The primary outcome (MSIS) and secondary PROMs, including quality of life, general health, and MS-related symptoms, showed modest but statistically significant improvements immediately after the intensive intervention phase, followed by attenuation during the subsequent 21-month maintenance phase. Although such attenuation may be interpreted as a loss of intervention benefit, stabilization itself represents a meaningful outcome in MS. Although most participants had relapsing-remitting MS, progression independent of relapse activity is an increasingly recognized feature of MS, already occurring from the earliest stages.^33,34^ Within this context, the absence of deterioration during follow-up may be considered clinically relevant. Without a control group, we cannot determine with certainty whether the observed stabilization differs from the expected natural disease course. Nevertheless, lifestyle interventions may serve as a complementary strategy not necessarily to reverse symptoms but to support resilience and slow functional decline, making sustained stabilization over time a meaningful outcome in its own right.

Long-term follow-up data on multimodal lifestyle interventions in MS are limited, but available studies consistently report modest direct effects with partial maintenance over time.^12,35-37^ Previous investigations on intensive, short-term lifestyle workshops focusing on diet, physical activity, stress management, and related behaviors have shown small improvements in the impact of MS disease, although these tend to diminish over time. By contrast, more lasting improvements have been observed in quality-of-life outcomes.^12,35^ Pilot studies further suggest short-term improvements in fatigue that tend to stabilize rather than continue to improve during longer-term follow-up.^36,37^ The present findings closely align with this overall pattern.

Although these changes are statistically significant at group level, only a limited proportion of participants exceeded the SDC. The most pronounced effects were observed for BMI with approximately 20% exceeding the SDC. This is particularly relevant given the increased risk of overweight and obesity in people with MS,^38^ which is associated with higher risk of comorbidities and greater disease severity.^39,40^

The SDC represents a conservative threshold reflecting changes beyond measurement error rather than clinical relevance.^41^ Unlike MIC thresholds, derived from different populations and measurement contexts, the SDC was calculated directly from our own data, providing a population-specific measure of reliable change. It is often stricter than the MIC, which aims to capture the smallest change perceived as beneficial by patients.^30^ Considering that MIC's are typically smaller than the SDC, it may be that some participants experienced meaningful changes that could not be detected using SDC. Indeed, published MIC thresholds for the MSIS-29 physical subscale (8.0 and 9.1 points) and psychological subscale (5.5 points) are smaller than the SDC in our study, indicating that individual MIC-level changes fall within the range of measurement error and should therefore be interpreted cautiously. Nonetheless, when applying MIC thresholds as reference, approximately 19%–25% of participants showed meaningful physical changes and 40%–45% exceeded psychological MIC (eTable 8). From a statistical perspective, approximately 2.5% of participants are expected to exceed the SDC due to random variation.^42^ Observed proportion exceeding SDC was only modestly higher than this expectation, indicating small individual-level effects. In a progressive disease such as MS, these change-based thresholds may not fully capture the value of stabilization. For patients in whom preventing deterioration is a meaningful outcome, failure to meet SDC or MIC thresholds should not be directly interpreted as evidence that an intervention is without benefit.

A notable finding was the presence of improvement during the run-in period for several outcomes, including the MSIS psychological, physical quality of life, fatigue, and perceived stress. These run-in improvement may reflect anticipation effects, increased health awareness after study enrollment, or early behavioral adjustments in response to monitoring and self-reflection and may resemble placebo-like or expectancy effects.^43^ Identifying such preintervention changes underscores the importance of accounting for baseline trajectories in lifestyle trials, as failure to do so may lead to overestimation or misattribution of intervention effects.

Among lifestyle behaviors, dietary adherence emerged as the most responsive outcome. Diet adherence improved substantially during the intensive intervention phase and, although partially attenuated during maintenance phase, remained significantly improved relative to baseline over the long term. This pattern was mirrored by modest but sustained improvements in BMI. These findings are consistent with previous long-term lifestyle studies in MS, in which dietary improvements similarly showed greater durability than MSIS-29.^12,35^ This may suggest that dietary behavior may be particularly amenable to change within structured lifestyle programs and that such changes can be partially maintained even when intervention intensity decreases.

By contrast, no meaningful changes were observed in physical activity adherence. Approximately one-third of participants met national physical activity guidelines at baseline, and this proportion remained stable throughout the study. This stability may partly reflect the limited sensitivity of a dichotomous threshold; even in the general Dutch population, fewer than half meet the guideline.^44^ Furthermore, evidence from previous digital physical activity interventions in MS suggests improvements of approximately 2–15 minutes of moderate physical activity per week in intervention groups,^45,46^ modest gains that may not be reflected in a dichotomous guideline-based outcome measure. It is also important to note that the primary aim of the physical activity pillar was to increase overall physical activity levels rather than necessarily achieving full adherence to national guidelines. Future studies may benefit from continuous, device-based physical activity measures, which may better capture change and reduce misclassification. In addition, people with MS face disease-specific barriers to physical activity, such as fatigue, mobility limitations, and fluctuating symptoms, which may necessitate more intensive, individualized, or supervised approaches to induce sustained behavioral change.^47^

The attenuation of effects observed during maintenance phase coincided with a transition from an intensive, interactive intervention phase to a less frequent and less interactive maintenance phase. Reduced intensity and coaching, and declining motivation may all have contributed to this pattern. Although attenuation is common in behavioral interventions, these findings highlight the importance of designing strategies that support long-term adherence, potentially through ongoing engagement, personalization, or integration into routine care.

A key strength of this study is that the LIMS program is implemented in routine clinical practice rather than a controlled research setting, making it one of the few lifestyle interventions in MS that has been successfully translated into real-world care. The program continues to be offered nationwide by the Dutch foundation Voeding Leeft! and the Dutch MS Foundation, underscoring its sustainability and practical relevance. This strong real-world embedding enhances the external validity, while also acknowledging an inherent selection bias, as participants self-enrolled and were therefore likely to be more motivated, an issue common to lifestyle and self-management interventions. Additional strengths include the accessibility of the digital intervention, large sample size, the extended follow-up period of 2 years, and the use of piecewise mixed-effects models that explicitly captured run-in, intervention, and follow-up phases. The conservative evaluation of individual-level change using SDC thresholds further strengthens the interpretability of the results.

Several limitations should also be considered. First, the observational and pragmatic design precludes causal inference, and in the context of long-term, multimodal lifestyle interventions, strict randomization, and control over all relevant behavioral components may not be feasible. Furthermore, although the piecewise mixed-effects model and explicit run-in phase partially mitigate this concern, regression to the mean cannot be fully ruled out, particularly given the self-enrolled study population and the absence of a control group. In addition, mixed-effects models accommodate missing data under a missing-at-random assumption, missing-not-at-random (MNAR) cannot be fully ruled out. Participants with poorer symptom trajectories or declining motivation may have been more likely to discontinue, potentially resulting in overestimation of long-term effects. The sensitivity analysis presented in eAppendix 2 and eTables 3 and 4 partially addresses this concern. Long-term estimates should therefore be interpreted with appropriate caution. Second, the study population was predominantly female and relatively highly educated, resulting in underrepresentation of men and individuals with lower educational levels and possibly limited computer literacy, which limits generalizability to more socioeconomically diverse populations. Third, attrition increased over time and engagement declined during the maintenance phase, potentially resulting in selective retention of more motivated participants. Whether specific program characteristics contributed to differential attrition remains a question for future research. Furthermore, adherence was operationalized as session attendance, and data on individual access to digital content were unavailable. Engagement with the program outside of scheduled sessions was not captured, which may have led to an underestimation of overall program engagement. Finally, outcomes were primarily based on PROMs, which may be subject to reporting and expectancy bias, particularly in a motivated population.

From a clinical perspective, the modest effects observed suggest that lifestyle interventions alone are unlikely to yield large symptomatic improvements in people with MS. Nevertheless, they may serve an important complementary role by supporting behavioral change, promoting self-management, and potentially contributing to the prevention of symptom deterioration over time. The LIMS program demonstrates that a fully digital, multimodal lifestyle intervention can be successfully implemented in routine clinical practice, which is a notable strength given that many lifestyle interventions remain confined to research settings and fail to translate into real-world care.

At the same time, there is clear room for further optimization. In particular, future iterations may benefit from a stronger focus on physical activity, the integration of more targeted behavioral change strategies, and approaches aimed at engaging underrepresented and less motivated groups, including men and individuals with lower educational levels, who may differ in both barriers and responsiveness to lifestyle interventions.

The exclusive reliance on PROMs reflects a current constraint of large-scale digitally delivered lifestyle research, where logistical barriers to objective assessment remain substantial. PROMs capture what is most meaningful to patients themselves.^48,49^ Future research should explore complementary measures such as digital biomarkers, alongside continued use of PROMs as primary end points. Furthermore, future research should use controlled designs to strengthen causal inference. In addition, emerging evidence suggests that lifestyle interventions may influence pathophysiologic mechanisms underlying MS, highlighting the importance of integrating biological outcomes alongside clinical and behavioral measures in future studies^2^

From a policy perspective, the integration of digital lifestyle support into standard MS care raises important questions regarding long-term health benefits and sustainability. Rigorous evaluations of cost-effectiveness will be essential to inform reimbursement and policy decisions, particularly given the potential for lifestyle interventions to reduce symptom burden and possibly modify aspects of disease progression.

In conclusion, the multimodal digital LIMS program resulted in modest improvements immediately after the intervention, with partial attenuation during a 21-month aftercare period; however, outcomes generally remained improved relative to baseline, and the absence of deterioration over 21 months may in itself be considered clinically meaningful in a progressive neurologic disease.

## Glossary

BMI: body mass index
DMT: disease-modifying therapy
LIMS: Lifestyle in Multiple Sclerosis
MIC: minimal important change
MS: multiple sclerosis
MSIS-29: MS Impact Scale–29
PROM: patient-reported outcome measure
SDc: smallest detectable change
